# Multiobjective Optimization Design of a Fractional Order PID Controller for a Gun Control System

**DOI:** 10.1155/2013/907256

**Published:** 2013-05-26

**Authors:** Qiang Gao, Jilin Chen, Li Wang, Shiqing Xu, Yuanlong Hou

**Affiliations:** ^1^School of Mechanical Engineering, Nanjing University of Science and Technology, Nanjing 210014, China; ^2^Research Institute, North Heavy Industries Group Corp. LTD, Inner Mongolia, Baotou 014033, China

## Abstract

Motion control of gun barrels is an ongoing topic for the development of gun control equipments possessing excellent performances. In this paper, a typical fractional order PID control strategy is employed for the gun control system. To obtain optimal parameters of the controller, a multiobjective optimization scheme is developed from the loop-shaping perspective. To solve the specified nonlinear optimization problem, a novel Pareto optimal solution based multiobjective differential evolution algorithm is proposed. To enhance the convergent rate of the optimization process, an opposition based learning method is embedded in the chaotic population initialization process. To enhance the robustness of the algorithm for different problems, an adapting scheme of the mutation operation is further employed. With assistance of the evolutionary algorithm, the optimal solution for the specified problem is selected. The numerical simulation results show that the control system can rapidly follow the demand signal with high accuracy and high robustness, demonstrating the efficiency of the proposed controller parameter tuning method.

## 1. Introduction

Gun control equipments (GCEs) have been extensively believed to be one of the key components of fire control systems (FCSs); the motion robustness and the motion accuracy of the gun barrel are regarded as the two main challenges associated with the developments of GCEs possessing excellent performances [1, 2]. The motion control of gun barrels is an ongoing topic due to certain extremely complicated segments with strong nonlinearities and uncertainties [3–5], such as the time-varying parameters induced by the varying working conditions, the random external applied loads and the complex friction forces between the cannon and the trunnion, and so forth. To eliminate these nonlinearities that induced negative effects, a dominant method is the application of the well-known proportional-integral-derivative (PID) control strategy [3, 4, 6]. However, due to the inherent nonlinearities existing in gun control systems as mentioned above, it is hard for the linear PID control strategy to achieve excellent control behaviors, and consequently the unsuitable PID controller has significantly limited the dynamic performances of the GCEs. Facing this dilemma, a more robustness and efficient control method should be further explored for the gun control systems.

Fractional order PID (FOPID), which was first proposed by Podlubny, is the extended version of conventional integer order PID (IOPID) [7]. FOPID possesses unique characteristics of infinite dimensions, memory effects, low sensitiveness to external disturbances, and so forth, when comparing with IOPID. Moreover, abundant dynamics, high robustness, and fine tracking accuracy of control systems can be obtained when FOPID is applied [8–10]. One of the most tough problems for practical applications of FOPID is the determination of the controller parameters, which highly influences the stability and tracking performances of servosystems. However, there are no universal methods for optimally determining these parameters due to the complexity of fractional order operations [11, 12]. Up to date, various tuning methods have been proposed. For practical applications, these methods could mainly be classified into two sorts, namely, frequency domain based methods [12, 13] and time domain based evolutionary optimization based methods [14–16]. In frequency domain, the loop-shaping based method has recently been proposed and updated by Luo and Chen [12], Li et al. [17], Luo and Chen [18], and Luo et al. [19]. In this method, the following three items are specified to achieve desired performances of the control system, namely, the gain crossover frequency, the phase margin, and the flat phase constraint at the specified crossover frequency to guarantee system robustness. However, this method could only be implemented on fractional order PI or PD control systems for the reason that the three constraint relations could only be used to determine three parameters. Therefore, this method would not be suitable for typical FOPID control systems which generally possess five unknown parameters to be tuned.

Recently, multiobjective optimization based tuning method has been proposed, and it will be a very promising method for designing optimal controllers, and various design objectives have been employed in both frequency domain and time domain [20, 21]. In this paper, a typical FOPID is employed for motion control of gun control system to enhance tracking accuracy and system robustness. To optimally determine the parameters of FOPID, an evolutionary optimization scheme (EOS) is proposed from loop-shaping perspective. The remainder of this paper could be summarized as follows. In Section 2, the physical model of the gun control system is developed; The basic principles of FOPID and the corresponding parameter determination principle are introduced in Section 3. The improved adaptive multiobjective differential evolution algorithm is then detailed in Section 4.  Section 5 conducts the numerical simulation, and the results are carefully discussed. The main conclusions of this paper are drawn in Section 6.

## 2. Modelling the AC Servosystem for GCEs

**Figure 1:**
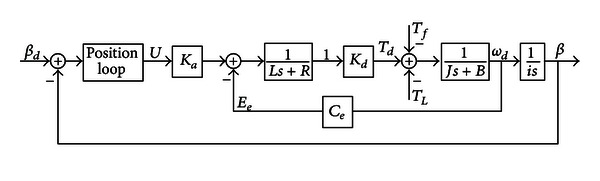
Schematic of the AC servosystem of GCEs.

The schematic of the AC servosystem utilized in certain sorts of GCEs is presented in Figure 1. Where *β*_*d*_ and *β* represent the desired angle position and the real angle position of the cannon, respectively. *U* is the control voltage; *K*_*a*_ is the amplify gain; *K*_*d*_ is the motor torque factor. *T*_*d*_, *T*_*L*_, and *T*_*f*_ are the motor torque, load torque disturbance, and friction torque disturbance, respectively. *R* and *L* represent the resistance and inductance of the motor armature, circuit, respectively. *E*_*e*_ is the counter-electromotive force (CEMF) of the motor armature and *C*_*e*_ denotes the CEMF coefficient. *J* is the total moment of inertia to the rotor; *B* is the viscous friction coefficient; *ω*_*d*_ is the angular velocity of the motor, *i* is the moderating ratio, and *s* denotes the Laplace operator.

Generally, the current time constant is much smaller than the mechanical time constant; the delay of the current response can be neglected and it yields

(1) $$\begin{array}{c} \frac{1}{Ls+R}=\frac{1}{R}\frac{1}{Ls/R+1}\approx \frac{1}{R}. \end{array}$$

The motor torque *T*_*d*_ is given as follows:

(2) $$\begin{array}{c} T_{d}=-\frac{K_{d}C_{e}}{R}\omega_{d}+\frac{K_{d}K_{a}}{R}U. \end{array}$$

According to the equilibrium equation of the torques, we can obtain

(3) $$\begin{array}{c} T_{d}-T_{L}-T_{f}=Ji\ddot{\beta }+Bi\dot{\beta }. \end{array}$$

Substituting (2) into (3) yields

(4) $$\begin{array}{c} Ji\ddot{\beta }+Bi\dot{\beta }=-\frac{K_{d}C_{e}}{R}\omega_{d}+\frac{K_{d}K_{a}}{R}U-T_{L}-T_{f}. \end{array}$$

When the motor torque and load torque disturbance are ignored, the govern principle of the AC servosystem can be obtained:

(5) $$\begin{array}{c} \ddot{\beta }+\left(\frac{B}{J}+\frac{K_{d}C_{e}}{JR}\right)\dot{\beta }=\frac{K_{d}K_{a}}{iJR}U. \end{array}$$

The transfer function of the AC servosystem could be obtained by taking Laplace transformation of (4), which could be given as

(6) $$\begin{array}{c} P\left(s\right)=\frac{\beta \left(s\right)}{U\left(s\right)}=\frac{K_{d}K_{a}}{i}\frac{1}{s\left(JRs+BR+K_{d}C_{\text{e}}\right)}. \end{array}$$

## 3. The Fractional Order PID Controller

### 3.1. A Preliminary to FOPID

According to the works of Podlubny, the PI^*μ*^D^*λ*^ controller is introduced, where *μ* and *λ* denote the order of an integrator and a differentiator, respectively. The control law of such a controller can be written as [7, 9, 22]

(7) $$\begin{array}{c} u\left(t\right)=k_{p}e\left(t\right)+k_{i}D_{t}^{-\mu }e\left(t\right)+k_{d}D_{t}^{\lambda }e\left(t\right),\\ D_{t}^{\alpha }e\left(t\right)={}_{0}D_{t}^{\alpha }e\left(t\right), \end{array}$$

where *k*_*p*_, *k*_*i*_, and *k*_*d*_ are proportion, integrator, and differentiator gain, respectively. _*t*_0__ *D*_*t*_^*α*^ *f*(*t*) is the noninteger order fundamental operator, and it is defined as [8, 23]

(8) $$\begin{array}{c} {}_{t_{0}}D_{t}^{\alpha }f\left(t\right)=\{\begin{array}{cc} \frac{d^{\alpha }}{dt^{\alpha }}, & ℜ\left(\alpha \right)>0\\ 1, & ℜ\left(\alpha \right)=0\\ \int_{t_{0}}^{t}{\left(d\tau \right)}^{\alpha }, & ℜ\left(\alpha \right)>0, \end{array} \end{array}$$

where *t*_0_ and *t* are the limits of the operation, and *α* is the order. 

Generally, there are two common definitions of the operator, known as Grunwald-Letnikov (G-L) definition and Riemann-Liouville (R-L) definition. The G-L definition is commonly utilized to directly conduct numerical computations, which is given as follows [9, 22]:

(9) $$\begin{array}{c} {}_{t_{0}}D_{t}^{\alpha }f\left(t\right)=h^{-\alpha }\sum_{j=0}^{\left[\left(t-t_{0}/h\right)\right]}b_{j}f\left(t-jh\right), \end{array}$$

where *b*_0_ = 1, *b*_*j*_ = [1 − (1 + *α*)/*j*]*b*_*j*−1_, *h* is calculation step. Substituting (9) into (7), the discrete FOPID control law can be obtained:

(10) u(k)=kpe(k)+kihs−μ∑j=0(t/h)bje(k−1)+kdhsλ∑j=0(t/h)qje(k−1).

As is discussed above, the FOPID has five parameters (*k*_*p*_, *k*_*i*_, *k*_*d*_, *μ*, *λ*) to be tuned. This adds more flexibility to controller design, and more dynamics behaviour can be obtained. However, this may simultaneously enhance the complexity in the selection of optimal control parameters.

By taking Laplace transformation of (7), the transfer function of FOPID could be obtained as

(11) $$\begin{array}{c} C\left(s\right)=k_{p}+\frac{k_{i}}{s^{\mu }}+k_{d}s_{}^{\lambda },\\ C\left(j\omega \right)=k_{p}+\frac{k_{i}}{{\left(j\omega \right)}^{\mu }}+k_{d}{\left(j\omega \right)}_{}^{\lambda }. \end{array}$$

Since *j* ^*α*^ = (*e* ^*j*(*π*/2)^)^*α*^ = cos⁡(*απ*/2) + *j*sin(*απ*/2), the transfer function of FOPID could be rewritten as

(12) C(jω)=[kp+kiωμcos⁡(π2μ)+kdωλcos⁡⁡(π2λ)] +j[kdωλsin(π2λ)−kiωμsin(π2μ)].

The phase and gain of the FOPID could be further given as

(13) $$\begin{array}{c} \left|C\left(j\omega \right)\right|=\sqrt{{\left[k_{p}+\frac{k_{i}}{\omega^{\mu }}\cos\left(\frac{\pi }{2}\mu \right)+k_{d}\omega^{\lambda }\cos\left(\frac{\pi }{2}\lambda \right)\right]}^{2}+{\left[k_{d}\omega^{\lambda }\sin\left(\frac{\pi }{2}\lambda \right)-\frac{k_{i}}{\omega^{\mu }}\sin\left(\frac{\pi }{2}\mu \right)\right]}^{2}},\\ \text{Arg}\left[C\left(j\omega \right)\right]=\arctan\frac{k_{d}\omega^{\lambda }\sin\left(\left(\pi /2\right)\lambda \right)-\left(k_{i}/\omega^{\mu }\right)\sin\left(\left(\pi /2\right)\mu \right)}{k_{p}+\left(k_{i}/\omega^{\mu }\right)\cos\left(\left(\pi /2\right)\mu \right)+k_{d}\omega^{\lambda }\cos\left(\left(\pi /2\right)\lambda \right)}. \end{array}$$

### 3.2. Frequency Domain Analysis of FOPID Gun Control System

As for the AC servosystem, the phase and gain of the plant in (6) can be given by

(14) $$\begin{array}{c} P\left(j\omega \right)=\frac{K_{d}K_{a}}{iJR}\frac{JR}{{\left(j\omega \right)}^{2}JR+\left(BR+K_{d}C_{e}\right)\left(j\omega \right)},\\ \left|P\left(j\omega \right)\right|=\frac{K_{d}K_{a}}{i\omega }\frac{1}{\sqrt{\omega^{2}{\left(JR\right)}^{2}-\left(BR+K_{d}C_{e}\right)}},\\ \text{Arg}\left[P\left(j\omega \right)\right]=-\frac{\pi }{2}+\arctan\frac{\left(BR+K_{d}C_{e}\right)}{JR\omega }. \end{array}$$

The transfer function of the open-loop control system can be given as

(15) $$\begin{array}{c} G\left(s\right)=P\left(s\right)C\left(s\right). \end{array}$$

The gain and phase of the open-loop system can be given as

(16) $$\begin{array}{c} \left|G\left(j\omega \right)\right|=\left|P\left(j\omega \right)C\left(j\omega \right)\right|,\\ \text{Arg}\left[G\left(j\omega \right)\right]=\text{Arg}\left[P\left(j\omega \right)\right]+\text{Arg}\left[C\left(j\omega \right)\right]. \end{array}$$

### 3.3. FOPID Design Specifications in Frequency Domain

Here, three specifications to be met by the FOPID controller are applied [12, 17–19]: phase margin specification, robustness to gain variations, and gain crossover frequency specification. To guarantee the robustness and stability of the control system, an extra-constrain, namely, output disturbance rejection capacity, is also employed [11]. The specifications and the constrain will be detailed below.

#### 3.3.1. Phase Margin Specification

Consider

(17) $$\begin{array}{c} \text{Arg}\left[P\left(j\omega_{c}\right)C\left(j\omega_{c}\right)\right]=-\pi +\phi_{m}, \end{array}$$

where *ω*_*c*_ is the gain crossover frequency interested, and *ϕ*_*m*_ is the phase margin required.

#### 3.3.2. Robustness to Gain Variations

Consider

(18) $$\begin{array}{c} {\frac{\text{dArg}\left[P\left(j\omega \right)C\left(j\omega \right)\right]}{\text{d}\omega }|}_{\omega =\omega_{c}}=0. \end{array}$$

With this condition, the phase Bode plot is flat at the gain crossover frequency. It means that the system is more robust to gain changes, and the overshoots of the response are almost the same.

#### 3.3.3. Gain Crossover Frequency Specification

At the gain crossover frequency point, the amplitude of the open-loop transfer function should be zero,

(19) $$\begin{array}{c} {\left|G\left(j\omega_{c}\right)\right||}_{\text{d}B}={\left|P\left(j\omega_{c}\right)C\left(j\omega_{c}\right)\right||}_{\text{d}B}=0. \end{array}$$

#### 3.3.4. Output Disturbance Rejection Capacity

A constraint on the sensitivity function *S* can be defined

(20) |S(jω)||dB=|11+P(jω)C(jω)||dB≤AdB,    ∀ω≥ωs rad/s,

with *A* the desired value of the sensitivity function for frequencies *ω* ≥ *ω*_*s*_ rad/s (desired frequency range).

### 3.4. Determination of Optimal Parameters

The three specifications may give constrains to parameter selection process; however, the five control parameters could not be optimally determined by (17)–(19). Thus, to solve this problem, a multiobjective optimization scheme is established in this paper to help the parameter determination process. In this scheme, the tuning process could be formulated as follows:

(21) min⁡ J1=|G(jωc)||dB+dArg[G(jω)]dω|ω=ωc+Arg[G(jωc)]+π−ϕm,         J2=|max⁡⁡(|S(jωi)|)|dB−AdB|, i=1,2,…5,s.t. 0<μ, λ<1, kp,ki,kd>0,

where, *ω*_*i*_ denotes selected frequency in the working bandwidth.

As it is evident in (21), it is a nonlinear multiobjective optimization problem. The complexity of this set of nonlinear equations is very significant, especially when fractional orders of differential operations are introduced, and finding out the analytical solution is not trivial. Heuristic optimization seeks good feasible solutions to a set of optimization problems in circumstances where the complexities of the problem or the limited time available for solution do not allow exact solution. It would be suitable to solve the complicated parameter determination problem faced in this paper. Motivated by this, an improved chaotic-self-adaptive multiobjective differential evolutionary algorithm is proposed for this problem, which will be detailed in the next section.

## 4. Improved Multiobjective Differential Evolutionary Algorithm

Differential evolution (DE) is an efficient evolutionary optimization algorithm motivated by natural selection. Compared with other evolutionary algorithms, DE is a simple yet powerful optimizer with fewer parameters and has much stronger ability in global searching. DE generates new offsprings by forming a noisy replica (trial vector) of each parent individual (target vector) of the population. The population is successfully improved by three basic operators: mutation, crossover, and selection [24–26]. 

As is discussed above, we shall often face problems with several objectives, and certain of them even contradict each other in that there is no single solution which simultaneously optimizes all functions. Instead, one has a set of optimal solutions. To solve the specified problems, there generally exist two efficient approaches. One is the weighted-sum approach where the multiobjective is converted to the single-objective problem by weighting sum of the objectives. This approach highly depends on decision-maker's preferences [27, 28]. Another approach is based on a set of solutions called Pareto-optimal solutions. This approach would be much more comprehensive and objective. Thus, the Pareto domain concept is embedded in DE to formulate a multiobjective differential evolution algorithm. To enhance robustness and adaptability of the algorithm for different optimization situations, an adapting law of the corresponding control parameters is also proposed. 

### 4.1. Definition of Pareto Dominance

Before discuss the employed MOCDE algorithm, the definition of Pareto dominance which is used to define Pareto-optimal points is firstly given as [29–31].

**Definition 1**

Let *x* = (*x*_1_, *x*_2_,…, *x*_*k*_) and *y* = (*y*_1_, *y*_2_,…, *y*_*k*_) be two vectors. Then, *x* dominates *y* (denoted by *x*≺*y*) if and only if (1) *x*_*i*_ ≤ *y*_*i*_ for all *i* = 1,…, *m*; and (2) *x*_*i*_ < *y*_*i*_ for at least one *i*.

**Definition 2**

We say that a vector of decision variables *x* ∈ *χ* ⊂ ℝ^*n*^ is nondominated with respect to *χ*, if there does not exist another *x*′ ∈ *χ* such that *f*(*x*′)≺*f*(*x*).

**Definition 3**

We say that a vector of decision variables x* ∈ *ℑ* ⊂ ℝ^*n*^ (*ℑ* is the feasible region) is Pareto optimal if it is nondominated with respect to *ℑ*.

We thus wish to determine the Pareto-optimal set from the set *ℑ* of all the decision variable vectors that satisfy Definitions 2 and 3.

### 4.2. Population Initialization

The initial population should better cover the entire search space as much as possible, so it is generated within the search space which is constrained by the prescribed lower and upper parameter bounds using the well known one-dimensional logistic chaotic map. The initial population is set as [24]

(22) $$\begin{array}{c} P^{0}=\left(x_{1}^{0,C},x_{2}^{0,C},x_{3}^{0,C},\ldots ,x_{N}^{0,C}\right), \end{array}$$

where the points *x*_*i*_^0,*C*^ are determined by

(23) $$\begin{array}{c} x_{i+1}^{0,C}=4x_{i}^{0,C}\left(1-x_{i}^{0,C}\right),\;x_{i}^{0,C}\in \left(1,0\right). \end{array}$$

As is known, the initial distribution features of the population will have significant effects on convergent characteristics; namely, an initial population covers area with better solutions will possess faster convergence. Motivated by this, Rahnamayan et al. proposed an *opposition based population initialization (OPI)* method for DE to enhance the convergent speeds [32]. In this paper, we extended this initialization concept for a multiobjective optimization process. The opposition partner of *x*_*i*_^0^ can be first expressed as [32, 33]

(24) $$\begin{array}{c} x_{i}^{0,\text{OP}}=u_{i}+l_{i}-x_{i}^{0,C}, \end{array}$$

where *u*_*i*_ and *l*_*i*_ denote the upper and lower boundary of the corresponding variable. 

By means of Pareto dominance concept, the better individuals of the initial population can be given as

(25) xi0=xi0,OP, if  xi0,OP≺xi0,C,xi0=xi0,C,  otherwise.

### 4.3. Mutation Operation

For each target vector *x*_*i*_^*k*^ at the *k*th generation, an associated mutant vector ${\hat{X}}^{k}=({\hat{x}}_{1}^{k},{\hat{x}}_{2}^{k},{\hat{x}}_{3}^{k},\ldots ,{\hat{x}}_{N}^{k})$ should be generated via certain mutation operators. In the process of mutation operation, the diversity of the population and the convergence rate of DE should be ensured. In conventional DE, the mutation operation will rely on the optimal individual of the last offspring to enhance the convergence rate. However, as for multiobjective optimization, there is no optimal individual, instead there will be a set of better individuals. So, a new mutation operation is proposed for the problem, which will be given as

(26) x^ik=xik+Fik(xdk−xik) +αFik(xnk−xr1k)+(1−α)Fik(xr2k−xr3k),

where *x*_*d*_^*k*^ and *x*_*n*_^*k*^ denote the randomly selected dominant solution and the nondomain solution in the parent populations in the *k*th generation for the *i*th individual, respectively. *x*_*ri*_^*k*^ denotes the randomly selected individual in the parent populations. *α* represents the weighting factor, *F*_*i*_^*k*^ denotes a mutation scale factor for the *i*th individual at the *k*th generation. In conventional DE, *F*_*i*_^*k*^ is a constant. To enhance the robustness and adaptability of the proposed algorithm, an adaptive scheme is employed for the determination of this factor. Thus, it can be expressed as [34]

(27) $$\begin{array}{c} F_{i}^{k}=\{\begin{array}{cc} F^{L}+\text{ran}{\text{d}}_{1}^{}\left(F^{U}-F^{L}\right), & \text{if}\;\;\tau <\text{ran}{\text{d}}_{2}^{}\\ F_{i}^{k-1}, & \text{otherwise}, \end{array} \end{array}$$

where *F* ^*U*^ and *F* ^*L*^ denote the preset upper and lower boundary of the factor.

### 4.4. Crossover Operation

After the mutation phase, the crossover operation is applied to each pair of the generated mutant vector ${\hat{X}}^{k}$ and its corresponding target vector *X* ^*k*^ to generate a trial vector [24]:

(28) $$\begin{array}{c} Y^{k}=\left(y_{1}^{k},y_{2}^{k},y_{3}^{k},\ldots ,y_{N}^{k}\right), \end{array}$$

(29) $$\begin{array}{c} y_{i}^{k}=\{\begin{array}{cc} y_{i}^{k}=x_{i}^{k}, & \text{rand}\;\;\left(\cdot \right)\leq C_{R}\\ {\hat{x}}_{i}^{k}, & \text{otherwise}, \end{array} \end{array}$$

where rand(·) is a randomly chosen real number in the range (0, 1) and CR is a user-specified crossover factor. 

### 4.5. Selection Operation

Evaluate the candidate *Y* ^*k*^ and its parent population *X* ^*k*^. There may exist three different conditions of the evaluation results; namely, the candidates dominate the parents and they are all the better ones. As for the selection operation, if the candidate dominates the parent, the candidate will replaces the parent. If the parent dominates the candidate, then the candidate will be discarded. Otherwise, the candidate is added in the population. If the population has more than PopSize individuals, truncate it according to the Filtrating Strategy proposed in [26]. 

## 5. Parameter Tuning Results and Discussion

### 5.1. Optimal Tuning of Controller Parameters

As for the description of the AC servosystem, the system parameters are chosen as follows: *J* = 0.0352 kg · m^2^, *K*_*d*_ = 0.195 N · m/A, *C*_*e*_ = 0.195 V/(rad · s^−1^), *i* = 315, *R* = 0.07 *Ω*, *B* = 0.000143 V/(rad · s^−1^). As for the optimization process, the population size is chosen as 30; the weighting factor *α* is chosen as 0.3; the upper and lower boundary of the mutation factor *F* ^*U*^ and *F* ^*L*^ are set as 0.9 and 0.3, respectively; and the crossover factor is chosen as 0.3. As for the design specifications of the controller, the interested crossover frequency is set as 4 Hz with respect to practical motions of the gun control servosystem. The required phase margin is set as *ϕ*_*m*_ = *π*/4; to describe the output disturbance rejection capacity of the control system, five specified frequencies within the range of 0.1 Hz to 4 Hz with constant interval are employed to formulate (21).

**Figure 2:**
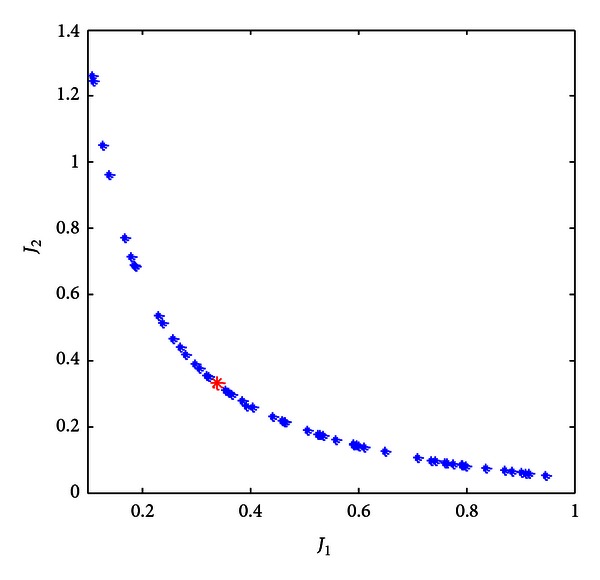
The Pareto-optimal solutions.

Figure 2 illustrates the Pareto-optimal solutions of this specified problem. It is evident that a compromise between the two performance requirements should be made, and the selected solution is highlighted in red color. As shown in Figure 2, the Pareto-optimal solutions have well-distributed spatial positions, demonstrating the efficiency of the employed *Filtrating Strategy *during the selection operation of the evolution process. The obtained optimal parameters of the controller corresponding to the optimal solution which is marked with red color in Figure 2 are *k*_*p*_ = 0.2246, *k*_*d*_ = 0.4652, *k*_*i*_ = 0.5681, *μ* = 0.8948, *λ* = 0.2162.

### 5.2. Performance Evaluation of the Optimal Control System

**Figure 3:**
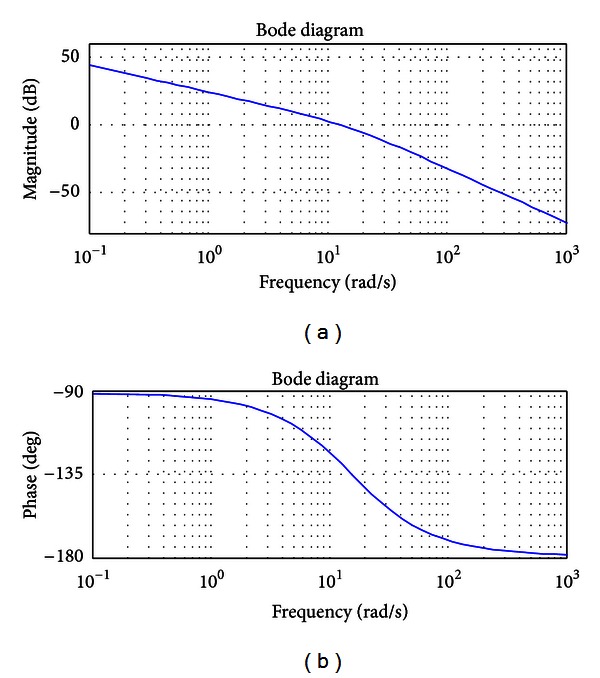
Bode diagram of *G*(*s*).

Figure 3 illustrates the Bode diagram of the open-loop control system. As is shown in Figure 3, the gain crossover frequency of the system is about 12.69 rad/s with a phase margin of 50.6°. A flat feature can be observed at the gain crossover frequency, and the change rate of the magnitude is about 0.13. It should be noticed that there exists a small shift of the desired performance due to the reason that a compromise has been made between the selected performances. In general, the obtained results in frequency domain validate efficiency of the proposed parameter determination process.

**Figure 4:**
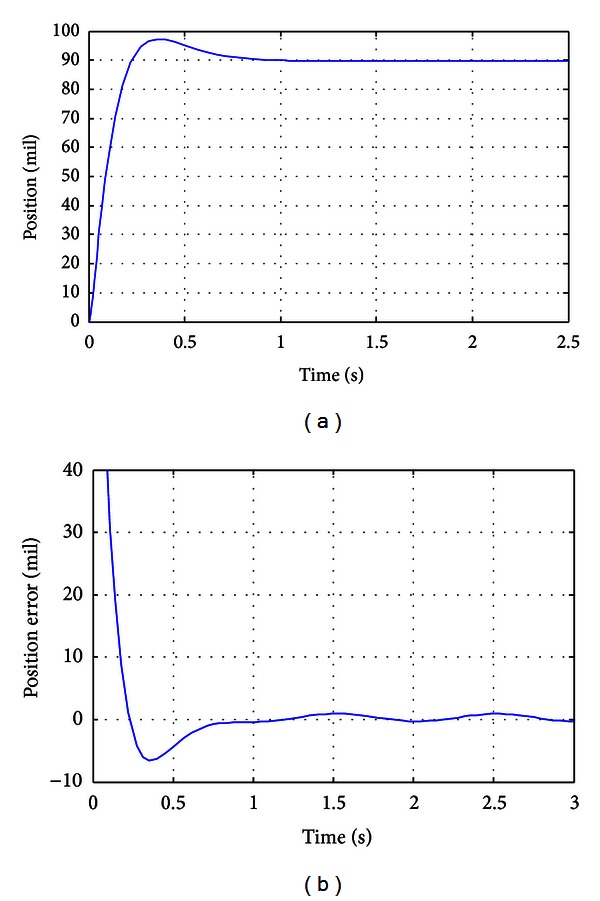
Step response of the control system.

To give a more comprehensive evaluation of the performances of the control system, detailed investigations in the time domain have been conducted.  Figure 4(a) illustrates the normal step response of the control system whereas the step value is set as 90 mil. To describe the robustness of the control system, a harmonic external disturbance with 7 mil amplitude and 0.5 Hz frequency is added to the control system; the corresponding positioning error is illustrated in Figure 4(b). From the response in Figure 4(a), the response time is about 0.23 s, and “zero” positioning error is obtained at about 0.88 s. A slight overshoot of about 7.78% is obtained. The results show that the control system can rapidly response to external demands, and fast petitioning criterion can be achieved. As is shown in Figure 4(b), the steady error of the system with external disturbance is about 1.1 mil, which is about 7.86% of the PV value of the disturbance. The results show that external disturbances can be well attenuated by the obtained optimal controller, demonstrating high robustness and strong disturbance rejection capacity of the control system.

**Figure 5:**
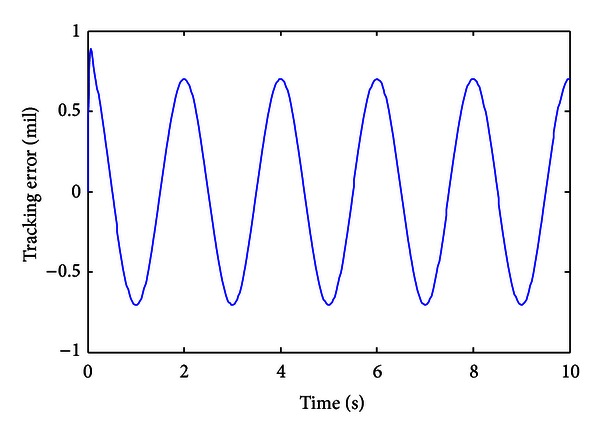
Tracking error of the control system.

To investigate the tracking performances of the control system, a harmonic signal with 90 mil amplitude and 1 Hz frequency is employed as the demand trajectory. To avoid repetition, Figure 5 just illustrates the tracking error of the control system. From the results shown in Figure 5, the tracking error is about 1.4 mil, which is about 0.78% of the full span of the demand signal. The tracking results show that the control system possesses high tracking accuracy, and it can be utilized for the high precision adjustment of the barrel of the gun.

## 6. Conclusion

In this paper, the fractional order PID control strategy is employed for a gun control system. To achieve optimal parameters of the controller, a multiobjective optimization scheme is developed from the loop-shaping perspective. To solve the nonlinear multiobjective optimization problem, a novel Pareto optimal solution based multiobjective differential evolution algorithm is proposed. To enhance the convergent rate of the optimization process, an opposition based learning method is embedded in the chaotic population initialization process. To enhance the robustness of the algorithm for different problems, an adapting scheme of the mutation operation is further employed. 

By means of numerical simulation, the Pareto fronts are obtained, and the corresponding optimal solution for the specified problem is selected. The results in frequency domain show that a compromise is well made between the selected objectives, demonstrating the efficiency of the proposed controller parameter tuning method. As for the investigations in time domain, the step response time of the control system is about 0.23 s and the tracking accuracy of the control system can reach up to 0.78% of the full span. Moreover, a strong attenuation of external disturbances can be obtained. All the results demonstrate that the control system can rapidly follow the demand signal with high accuracy and high robustness, and it will be very promising for engineering practices.
